# Identifying and revealing different brain neural activities of cognitive subtypes in early course schizophrenia

**DOI:** 10.3389/fnmol.2022.983995

**Published:** 2022-10-03

**Authors:** Tiannan Shao, Weiyan Wang, Gangrui Hei, Ye Yang, Yujun Long, Xiaoyi Wang, Jingmei Xiao, Yuyan Huang, Xueqin Song, Xijia Xu, Shuzhan Gao, Jing Huang, Ying Wang, Jingping Zhao, Renrong Wu

**Affiliations:** ^1^Department of Psychiatry, and National Clinical Research Center for Mental Disorders, The Second Xiangya Hospital of Central South University, Changsha, China; ^2^Department of Psychiatry, The First Affiliated Hospital of Zhengzhou University, Zhengzhou, China; ^3^Department of Psychiatry, Affiliated Nanjing Brain Hospital, Nanjing Medical University, Nanjing, China

**Keywords:** schizophrenia, cognitive subtypes, latent profile analysis, MCCB, MRI, ALFF

## Abstract

**Background:**

Cognitive subtypes of schizophrenia may exhibit different neurobiological characteristics. This study aimed to reveal the underlying neurobiological features between cognitive subtypes in the early course of schizophrenia (ECS). According to prior studies, we hypothesized to identify 2–4 distinct cognitive subtypes. We further hypothesized that the subtype with relatively poorer cognitive function might have lower brain spontaneous neural activity than the subtype with relatively better cognitive function.

**Method:**

Cognitive function was assessed by the MATRICS Consensus Cognitive Battery (MCCB). Resting-state functional magnetic resonance imaging scanning was conducted for each individual. There were 155 ECS individuals and 97 healthy controls (HCs) included in the subsequent analysis. Latent profile analysis (LPA) was used to identify the cognitive subtypes in ECS individuals, and amplitude of low-frequency fluctuations (ALFFs) was used to measure brain spontaneous neural activity in ECS individuals and HCs.

**Results:**

LPA identified two cognitive subtypes in ECS individuals, containing a severely impaired subtype (SI, *n* = 63) and a moderately impaired subtype (MI, *n* = 92). Compared to HCs, ECS individuals exhibited significantly increased ALFF in the left caudate and bilateral thalamus and decreased ALFF in the bilateral medial prefrontal cortex and bilateral posterior cingulate cortex/precuneus (PCC/PCu). In ECS cognitive subtypes, SI showed significantly higher ALFF in the left precentral gyrus (PreCG) and lower ALFF in the left PCC/PCu than MI. Furthermore, ALFFs of left PreCG were negatively correlated with several MCCB cognitive domains in ECS individuals, while ALFF of left PCC/PCu presented opposite correlations.

**Conclusion:**

Our findings suggest that differences in the brain spontaneous neural activity of PreCG and PCC/PCu might be the potential neurobiological features of the cognitive subtypes in ECS, which may deepen our understanding of the role of PreCG and PCC/PCu in the pathogenesis of cognitive impairment in schizophrenia.

## Introduction

Cognitive impairment is a core component of schizophrenia, which exists not only in the acute phase but also in the ultra-high risk stage of psychosis and persists during the clinical remission of psychiatric symptoms (Keefe et al., 2006; Bora and Murray, 2014). In schizophrenia, cognitive impairment is manifested in multiple domains, including verbal and visual learning, working memory, processing speed, problem solving, attention, and executive function (Nuechterlein et al., 2004; Sheffield et al., 2018), which could contribute to poor functional outcomes (Green, 2016). Although there is a generalized cognitive deficit in schizophrenia, not all individuals exhibit the same pattern of cognitive deficits, i.e., degrees of impairment in different cognitive dimensions from different sample groups are manifested differently (Carruthers et al., 2019). These heterogeneous patterns of cognitive impairment make it difficult to understand the pathophysiology of schizophrenia.

Accumulating evidence has successfully classified individuals with psychiatric disorders with similar cognitive characteristics and compared differences between distinct cognitive features. Participants could be classified according to their current cognitive function scores based on artificial classification (Ammari et al., 2010; Ortiz-Gil et al., 2011), or could be classified by data-driven approaches (Lim et al., 2020; Smucny et al., 2020). Instead of appearing to be limited to dividing participants into relatively intact or cognitively impaired subtypes by prior defined classification criteria, the data-driven approach could provide valuable insight into multiple cognitive subtypes that exist in psychiatric disorders (Carruthers et al., 2019).

Abnormal cognitive function also has relevant neuroimaging features. Growing attention has been paid to identifying the neurobiological changes of cognitive subtypes in schizophrenia through neuroimaging (Van Rheenen et al., 2018; Lewandowski et al., 2019). Several studies using data-driven methods have illustrated the different patterns of brain structure or resting-state functional connectivity among cognitive subtypes in individuals with schizophrenia. For brain structure, near-normal cognitive groups and impaired cognitive groups of individuals with schizophrenia or schizoaffective disorders were found at varying levels of cortex thickness (Cobia et al., 2011), or different gray and white matter volumes (Wexler et al., 2009; Van Rheenen et al., 2018). For brain function connectivity networks, three cognitive subtypes classified by *k*-means clustering method from 67 individuals with schizophrenia spectrum disorders showed unique hyper- or hypo-connectivity in specific functional networks (Rodriguez et al., 2019). However, previous neuroimaging studies have used structural measurements and functional connectivity approaches to investigate the neurobiological features between cognitive subtypes in schizophrenia; to the best of our knowledge, there was no study investigating differences in brain spontaneous neural activity between cognitive subtypes of ECS yet.

The amplitude of low-frequency fluctuations (ALFFs) is often used to characterize brain spontaneous neural activity (Zang et al., 2007). Several studies have investigated the association between ALFF and cognitive function in schizophrenia (Zhou et al., 2014; Wang et al., 2019). Therefore, the purpose of this study was to determine the differences in brain spontaneous neuronal activity measured by ALFF between cognitive subtypes of individuals with ECS. We compared the cognitive subtypes derived from latent profile analysis (LPA) with healthy controls (HCs) to assess the condition of cognitive impairment (Miettunen et al., 2016). Based on two recent systematic reviews on cognitive subtypes in schizophrenia, we expected to find 2–4 distinct cognitive subtypes: a severely impaired subgroup, 1–2 moderately impaired subgroups, and a relatively intact cognitive subgroup (Carruthers et al., 2019, 2021). We further hypothesized that cognitive subtypes might exhibit different brain spontaneous neuronal activities, and the brain spontaneous neuronal activity may be lower in the subtype with poorer cognitive function than in the subtype with better cognitive function.

## Methods

### Participants

We recruited ECS individuals and HCs from three clinical medical centers, the Second Xiangya Hospital of Central South University (Center 1), the Affiliated Nanjing Brain Hospital of Nanjing Medical University (Center 2), and the First Affiliated Hospital of Zhengzhou University (Center 3). All recruited ECS individuals met the criteria of the Diagnostic and Statistical Manual of Mental Disorders, Fourth Edition (DSM-IV) based on a Structured Clinical Interview for DSM-IV Axis-I Disorder (First et al., 1997), which were assessed by two well-trained psychiatrists in each center.

For ECS individuals, the further inclusion criteria were as follows: (1) aged 16–60; (2) experienced the first episode of psychiatric symptoms or illness duration within 3 years but in the acute phase currently; (3) Positive and Negative Syndrome Scale (PANSS) total score >60. The exclusion criteria were: (1) comorbidity of physical diseases, or other psychiatric disorders that met the DSM-IV criteria; (2) comorbidity of substance abuse or addiction; (3) unable to complete cognitive function tests and magnetic resonance imaging (MRI) examinations; (4) pregnant or lactating women. In addition, HCs with first-degree relatives that had any psychiatric disorders were also excluded.

Written informed consents were obtained from all participants or first-degree relatives of ECS individuals before participating in the study. This study was approved by the Ethics Committee of the Second Xiangya Hospital of Central South University, the Ethics Committee of the Affiliated Nanjing Brain Hospital of Nanjing Medical University, and the Ethics Committee of the First Affiliated Hospital of Zhengzhou University.

Our study totally enrolled 181 ECS individuals and 107 HCs. After neuroimaging preprocessing, 155 ECS individuals and 97 HCs were included in the subsequent analysis (subjects with head motion exceeding 2 mm or head rotation exceeding 2° were excluded). Among 155 ECS individuals, 119 (76.8%) individuals received risperidone, 16 (10.3%) olanzapine, 11 (7.1%) amisulpride, seven (4.5%) aripiprazole, and two (1.3%) paliperidone. The doses of antipsychotic drugs were equivalent to chlorpromazine dose by the defined daily doses (DDDs) method (Leucht et al., 2016). The remaining subjects of each center were as follows: 34 ECS individuals and 18 HCs (Center 1); 38 ECS individuals and 36 HCs (Center 2); 83 ECS individuals and 43 HCs (Center 3). Demographic and clinical data of ECS individuals and HCs from three centers are shown in Supplementary Table 1.

### Cognitive assessment

We used Measurement and Treatment Research to Improve Cognition in Schizophrenia Consensus Cognitive Battery (MCCB) to evaluate cognitive function (Kern et al., 2008; Nuechterlein et al., 2008). The MCCB was widely used and translated into Chinese version (Shi et al., 2015). It includes nine tasks across seven cognitive domains: (1) speed of processing (Trail Making Test, part A; Brief Assessment of Cognition in Schizophrenia, Symbol Coding; Category Fluency Test, Animal); (2) attention and vigilance (Continuous Performance Test-Identical Pairs); (3) working memory (Wechsler Memory Scale, spatial span); (4) verbal learning (Hopkins Verbal Learning Test-Revised); (5) visual learning (Brief Visuospatial Memory Test); (6) reasoning and problem solving (Neuropsychological Assessment Battery, mazes test); (7) social cognition (Mayer–Salovey–Caruso Emotional Intelligence Test, managing emotions test). It takes 1–1.5 h for each subject to finish MCCB. Raw scores were converted to scale scores, then to Chinese-normalized *T* scores. *T* scores of seven cognitive domains and composite scores (the average *T* score of nine tasks) were calculated. In this study, all ECS individuals and HCs completed the MCCB.

### Neuroimaging data acquisition

The thorough description of this section is described in the Supplementary material.

### Neuroimaging data preprocessing

Image preprocessing was performed by the Data Processing & Analysis of Brain Imaging toolbox (DPABI, V4.2, http://rfmri.org/dpabi) running on MATLAB software (The MathWorks, Inc., Natick, MA, USA; Yan et al., 2016). We removed the first 10 scanning volumes in order to stabilize the magnetic resonance signal and reduce the impact of subjects not adapting to the scanning environment. Slice timing and head motion were corrected for each subject, and those whose head motion exceeded 2 mm or head rotation exceeded 2° were excluded. Afterward, images were spatially normalized to the standard Montreal Neurological Institute template by using warping parameters estimated from T1 images with a resampling standard voxel size of 3 mm × 3 mm × 3 mm. We used a 6 mm full-width at half-maximum Gaussian kernel to spatially smooth images and performed linear detrending. Then the nuisance signals were regressed out, including head motion effects (Friston 24-parameter model; Friston et al., 1996), white matter, and cerebrospinal fluid. Finally, band-pass filtering (0.01–0.08 Hz) was applied for the time series of each voxel to remove the effects of very-low-frequency drifts and high-frequency noise (Zang et al., 2007). After that, the time series of each voxel for each subject was transformed into the frequency domain under a fast Fourier transformation way in order to get the power spectrum. To standardize the ALFF values, the ALFF of each voxel would be converted into *Z* scores by subtracting the global mean and then dividing the global standard deviation.

### Multi-site effect harmonization

Before statistical analysis, we used the ComBat Harmonization method (http://github.com/Jfortin1/ComBatHarmonization) for preprocessed data to eliminate the inter-site effects (Fortin et al., 2017). This widely used method could effectively remove unwanted variation introduced by the site, and increase statistical power (Fortin et al., 2018; Radua et al., 2020). In addition, group, age, sex, and education level were protected during the removal of inter-site effects.

### Latent profile analysis

Latent profile analysis is used to classify individuals into heterogeneous subtypes based on latent variable models. It could explain the associations between the observed continuous indicator variables by regressing the continuous indicator variables onto a set of one or more latent class variables (Miettunen et al., 2016). LPA is a model-based approach, and thus has fewer prerequisites for application, more reasonable clustering criteria and result testing, and less arbitrariness than traditional clustering methods (e.g., *k*-means; Brusco et al., 2017; Schreiber, 2017). The flexibility of LPA makes it adaptable to the heterogeneous study of complex psychiatric and psychological phenomena with effective classification of cognitive subtypes (Lim et al., 2020; Smucny et al., 2020; De Meo et al., 2021).

Latent profile analysis was conducted by Mplus version 7.11 to identify potential homogenous subtypes of ECS individuals based on cognitive performance in seven MCCB cognitive domains (Muthén and Muthén, 2015). The number of classes was determined from an examination of models fit statistics rather than hypothesized. These model fit indices included log-likelihood ratio (LLR; Woolf, 1957), Akaike's information criteria (AIC; Akaike, 1987), Bayesian information criteria (BIC; Schwarz, 1978), sample-size adjusted BIC (ABIC; Sclove, 1987), and entropy (Celeux and Soromenho, 1996). Lo-Mendell-Rubin (LMR) tests and bootstrapped likelihood ratio tests (BLRTs) were also conducted to evaluate the significance of model improvement between *n* and *n* – 1 number of classes (McLachlan, 1978; Lo et al., 2001). A total of five models were estimated specifying from 1 to 5 latent classes.

### Statistical analysis

Once the potential subtypes were identified, group differences in demographic data, clinical data, PANSS scores, and MCCB scores were analyzed by SPSS version 22.0 (IBM, Armonk, NY, USA) by using one-way analysis of variance (ANOVA), two-sample *t*-test, or chi-squared test. Furthermore, eta-squared (η^2^) was used to calculate the effect size of comparisons of each MCCB cognitive domain (Cohen, 1988). Post-hoc comparisons were carried out by Bonferroni correction if ANOVA showed significant differences between subtypes. Generally, *p*-values of < 0.05 were accepted as statistically significant.

ALFF analysis was conducted in the Statistical Parametric Mapping 12 toolbox (SPM12, https://www.fil.ion.ucl.ac.uk/spm/software/spm12). Two-sample *t*-test was designed for the comparison of ALFF maps between ECS individuals and HCs, and in ECS cognitive subtypes, with age, sex, education, and mean frame-wise displacement Jenkinson as covariates. In addition, PANSS total score was also controlled for subtypes comparison to investigate whether the result was consistent after subtracting out the effect of symptom severity. Multiple comparisons were corrected using the cluster-wise family-wise error (FWE) rates correction (cluster-wise FWE *p* < 0.05) with a combined individual voxel threshold of *p* < 0.001. Significant brain regions with discrepant ALFF between ECS cognitive subtypes were regarded as regions of interest (ROI) to extract ALFF values for subsequent correlation analysis in SPSS. To further explore the specific associations between ALFF in ROIs and MCCB scores, their correlations coefficient between ALFF values in ROIs and MCCB scores in ECS and HCs were calculated, respectively. Pearson's *r* was used to calculate the effect size of correlations between ALFF in ROIs and MCCB cognitive scores (Cohen, 1988). The significant level of correlations was corrected by the false discovery rate (FDR) at *q* < 0.05.

## Results

### LPA results based on MCCB cognitive domains

According to the results of model estimation, the 2-class solution presented a better fit than the 1-class solution. Though the other solutions suggested better fits than the 2-class solution based on AIC, BIC, and ABIC, there was no significant improvement over the 2-class solution according to the *p*-value of LMR and the *p*-value of BLRT (Table 1, Supplementary Figure 1). The 2-class solution classified 40.6% of the ECS individuals into the severely impaired subtype (SI, *n* = 63) and 59.4% into the moderately impaired subtype (MI, *n* = 92), respectively.

**Table 1:** Model estimations of latent profile analysis based on MCCB cognitive domains.

**Classes**	**LLR**	**AIC**	**BIC**	**ABIC**	**Entropy**	***p*-value of LMR**	***p*-value of BLRT**
1	−4208.287	8444.573	8487.181	8442.868	–	–	–
2	−4093.591	8231.181	8298.137	8228.502	0.822	< 0.001	< 0.001
3	−4065.038	8190.077	8281.379	8186.422	0.808	0.498	< 0.001
4	−4047.426	8170.852	8286.502	8166.223	0.798	0.132	< 0.001
5	−4032.352	8156.704	8296.701	8151.101	0.781	0.759	< 0.001

LLR, log-likelihood ratio; AIC, Akaike's information criteria; BIC, Bayesian information criteria; ABIC, sample-size adjusted Bayesian information criteria; LMR, Lo-Mendell-Rubin test; BLRT, bootstrap likelihood ratio test.

### Demographic and clinical characteristics

**Figure 1:**
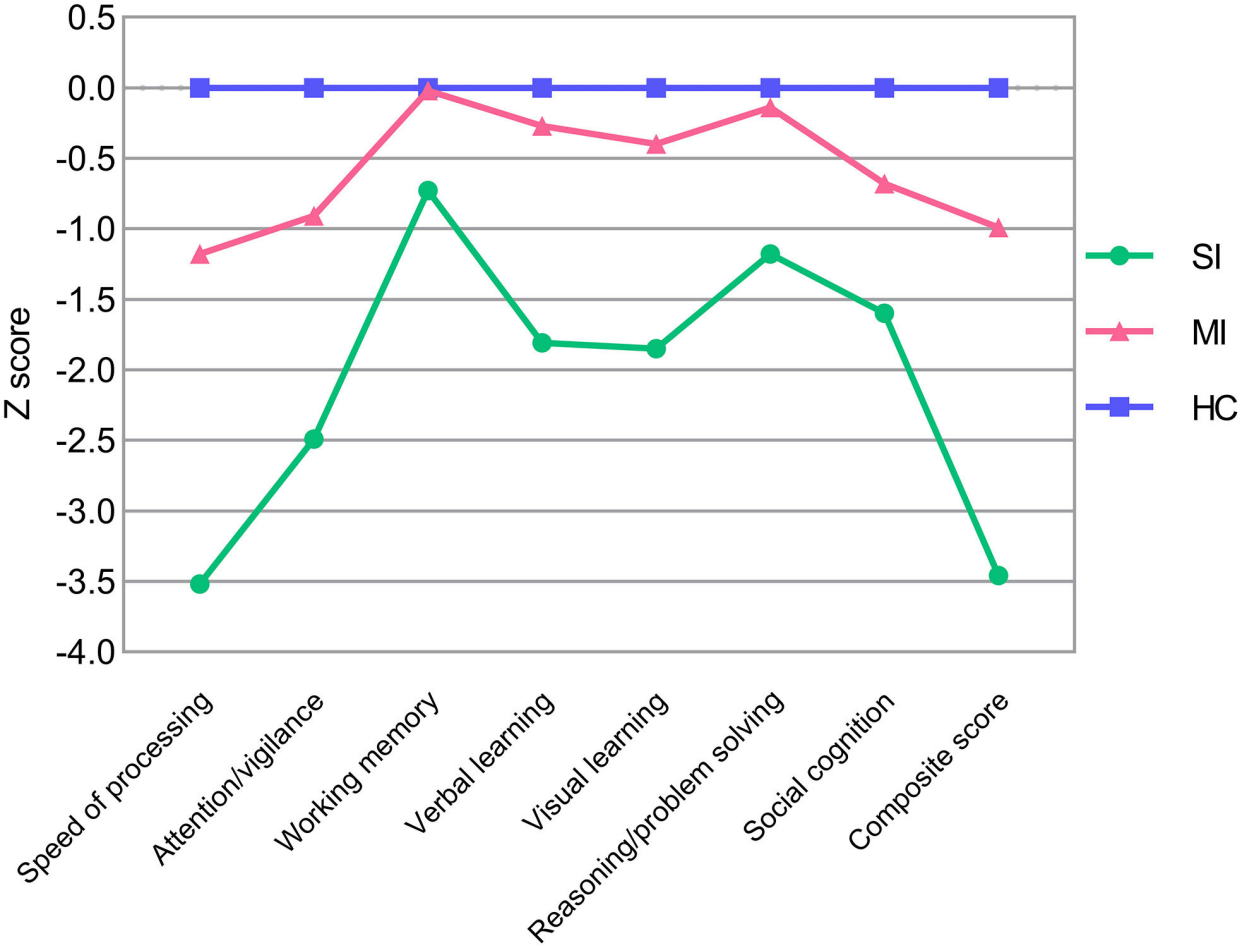
MCCB cognitive scores in ECS cognitive subtypes (SI and MI) and HCs. *Z* scores of ECS cognitive subtypes are standardized against means and SDs of HC (mean = 0, SD = 1). *Abbreviations:* ECS, early course schizophrenia; SI, severely impaired subtype; MI, moderately impaired subtype; HC, healthy control; MCCB, MATRICS Consensus Cognitive Battery; SD, standard deviation.

Demographic and clinical characteristics of two ECS cognitive subtypes and HCs are shown in Table 2. The sex composition of SI significantly differed from MI. Besides, education levels were similar in both subtypes but significantly lower than the HCs. For symptom severity, PANSS scores of SI were significantly higher than MI except for positive score. Significant pairwise differences in MCCB domains were exhibited among the three groups, while differences in the working memory domain and reasoning/problem-solving domain between MI and HCs were not significant (Table 2). In addition, the MCCB performance of HC were taken as the norm to standardize the cognitive score of ECS into *Z* scores (see Figure 1 and Supplementary Table 2).

**Table 2:** Demographic and clinical data of ECS cognitive subtypes and HCs.

	**SI (*n* = 63)**	**MI (*n* = 92)**	**HC (*n* = 97)**	***F*/χ^2^*/t***	***p***	**Effect size (η^2^)**	***Posthoc* ^a^**
Age, years	23.84 (7.06)	25.38 (8.37)	25.03 (5.42)	0.944	0.391	–	–
Sex, female/male	26/37	59/33	54/43	7.918	0.019	–	SI ≠ MI, SI = HC, MI = HC
Education, years	11.24 (2.56)	11.32 (2.97)	13.49 (3.27)	16.255	< 0.001	–	SI, MI < HC
Duration, months	8.10 (10.09)	10.09 (10.23)	–	−1.197	0.233	–	–
CPZ-DDD, mg	254.92 (61.80)	278.15 (123.24)	–	–**1**.546	0.124	–	–
**PANSS**
Positive symptoms	23.38 (5.41)	21.90 (6.27)	–	1.523	0.130	–	–
Negative symptoms	27.06 (6.14)	22.25 (5.91)	–	4.903	< 0.001	–	–
General psychopathology	46.60 (6.82)	43.90 (6.31)	–	2.531	0.012	–	–
Total score	97.05 (13.19)	88.26 (12.54)	–	4.195	< 0.001	–	–
**MCCB**
Speed of processing	20.61 (7.74)	37.86 (7.17)	46.59 (7.38)	236.768	< 0.001	0.655	SI < MI < HC
Attention/vigilance	25.51 (11.09)	40.15 (10.81)	48.55 (9.25)	95.440	< 0.001	0.434	SI < MI < HC
Working memory	32.16 (9.82)	41.07 (9.41)	41.27 (12.40)	16.586	< 0.001	0.118	SI < MI, HC
Verbal learning	27.57 (8.31)	41.73 (8.60)	44.25 (9.23)	75.085	< 0.001	0.376	SI < MI < HC
Visual learning	28.38 (13.39)	44.33 (10.29)	48.77 (11.01)	63.938	< 0.001	0.339	SI < MI < HC
Reasoning/problem solving	28.87 (7.49)	39.86 (11.64)	41.37 (10.63)	31.027	< 0.001	0.199	SI < MI, HC
Social cognition	30.54 (9.60)	39.67 (10.85)	46.45 (9.98)	46.427	< 0.001	0.272	SI < MI < HC
Composite score	26.10 (4.89)	40.04 (4.88)	45.60 (5.63)	274.783	< 0.001	0.688	SI < MI < HC

Values are presented as mean (SD). SI and MI represent the two cognitive subtypes in ECS.

a Post-hoc comparisons were conducted using Bonferroni correction.

ECS, early course schizophrenia; SI, severely impaired subtype; MI, moderately impaired subtype; HC, healthy control; CPZ-DDD, chlorpromazine-defined daily dose; MCCB, MATRICS Consensus Cognitive Battery; PANSS, Positive and Negative Syndrome Scale; SD, standard deviation.

### ALFF differences between groups

**Figure 2:**
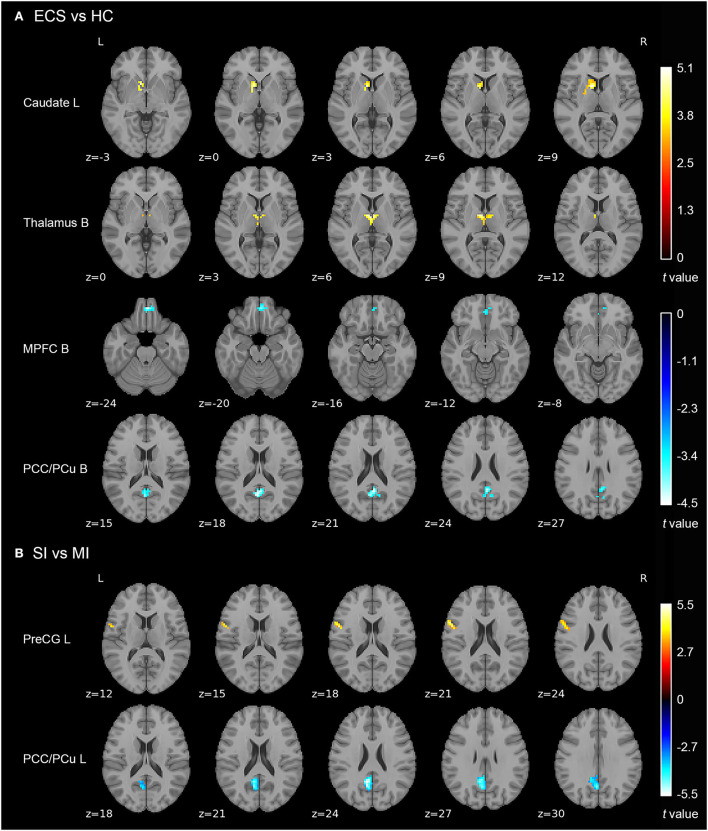
ALFF differences in ECS, HCs, and ECS cognitive subtypes (SI and MI). **(A)** Abnormal ALFF in ECS relative to HCs. **(B)** Increased ALFF in left PreCG and decreased ALFF in left PCC/PCu in SI relative to MI. Results were cluster-wise FWE corrected. *Abbreviations:* ALFF, amplitude of low-frequency fluctuations; ECS, early course schizophrenia; SI, severely impaired subtype; MI, moderately impaired subtype; HC, healthy control; MPFC, medial prefrontal cortex; PCC/PCu, posterior cingulate cortex/precuneus; PreCG, precentral gyrus; L, left; B, bilateral; FWE, family-wise error.

Results of the independent two-sample *t*-test (cluster-wise FWE *p* < 0.05) and cluster information are shown in Table 3 and Figure 2. Compared with HCs, ECS exhibited higher ALFF in the left caudate and bilateral thalamus, while lower ALFF was observed in the bilateral medial prefrontal cortex (MPFC) and bilateral posterior cingulate cortex/precuneus (PCC/PCu). For the subtypes comparison, ALFF of the left precentral gyrus (PreCG) was significantly higher in SI. On the contrary, the ALFF of left PCC/PCu in SI was significantly lower than MI. To control for the effect of PANSS and medication effects (chlorpromazine dose equivalence), we conducted the additional analysis with PANSS total score and medication effects as covariates. This result was similar to the previous result without controlling for the two covariates (see Supplementary material).

**Table 3:** Brain regions with ALFF differences in ECS and HCs and in ECS cognitive subtypes.

	**Brain regions**	**Cluster size**	**Peak coordinate (mm)*^a^***			**Peak *t*-value**
			***x***	***y***	***z***	
ECS > HC	Caudate L	98	−9	9	0	5.0849
	Thalamus B	54	−9	−6	9	4.7638
ECS < HC	MPFC B	49	3	48	−24	−4.4362
	PCC/PCu B	100	3	−54	21	−4.5327
SI > MI	PreCG L	47	−56	5	19	4.9245
SI < MI	PCC/PCu L	142	−6	−57	26	−5.488

SI and MI represent the two cognitive subtypes in ECS.

a Peak coordinate refers to the peak voxel location of the significant cluster in the Montreal Neurological Institute space.

ALFF, amplitude of low-frequency fluctuations; ECS, early course schizophrenia; SI, severely impaired subtype; MI, moderately impaired subtype; HC, healthy control; MPFC, medial prefrontal cortex; PCC/PCu, posterior cingulate cortex/precuneus; PreCG, precentral gyrus; L, left; B, bilateral.

### Correlations between ALFF of ROIs and cognition

**Figure 3:**
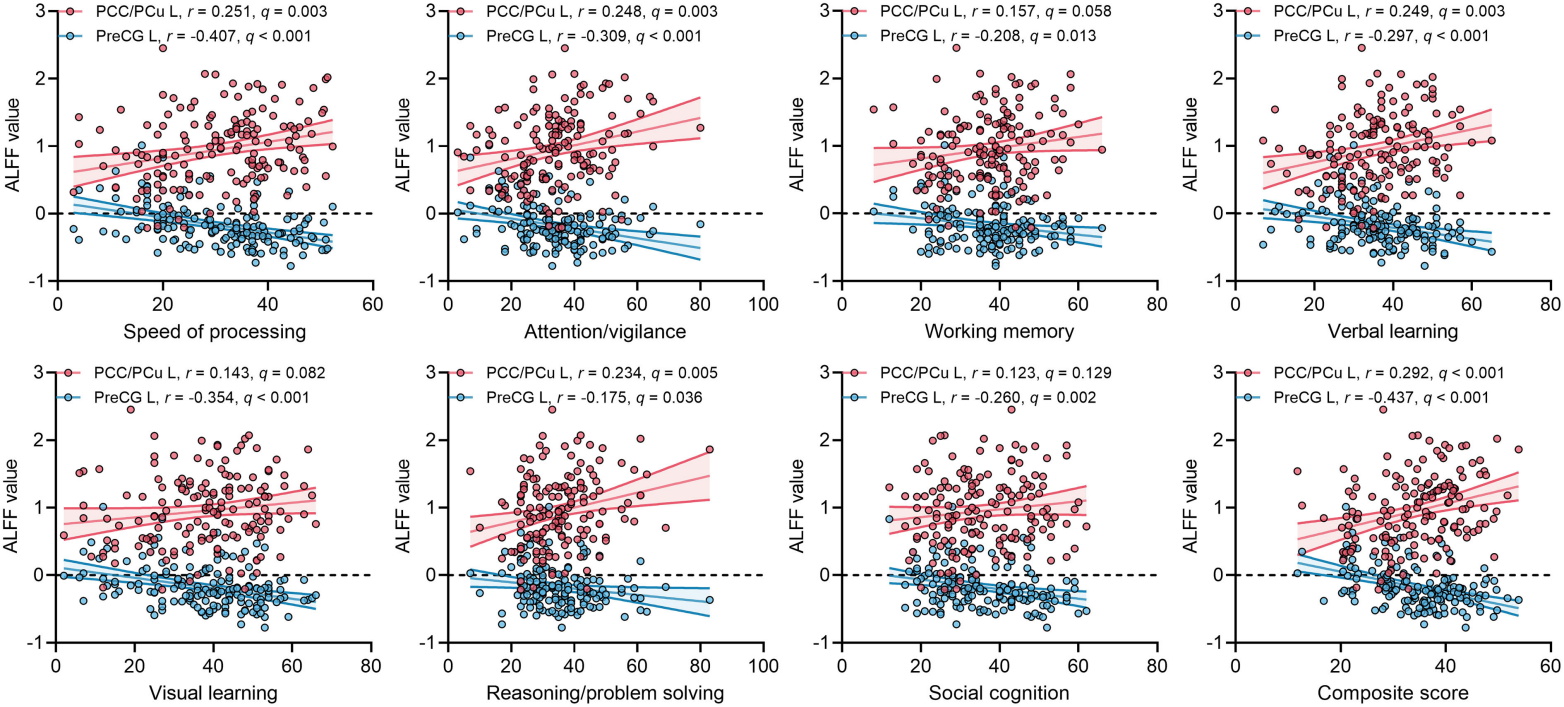
Correlations between ALFF values of two ROIs and MCCB cognitive scores in ECS. *q, p*-values corrected by FDR correction. *Abbreviations:* ALFF, amplitude of low-frequency fluctuations; ECS, early course schizophrenia; MCCB, MATRICS Consensus Cognitive Battery; PCC/PCu, posterior cingulate cortex/precuneus; PreCG, precentral gyrus; ROI, region of interest; FDR, false discovery rate; L, left.

For ECS individuals, there were significantly positive correlations between ALFF values of left PCC/PCu with MCCB speed of processing, attention/vigilance, verbal learning, reasoning/problem solving, and composite score (Figure 3). The effect sizes of the correlations were small to moderate. Moreover, ALFF in left PreCG was significantly negatively correlated with MCCB seven cognitive domains and composite score (Figure 3). Importantly, some correlations of ALFF in left PreCG had moderate to large effect sizes. No significant correlations were found in HCs.

## Discussion

The present study revealed the relationship between cognitive impairment and neurobiology in ECS individuals grouped according to their different patterns of cognitive deficit using a data-driven approach. We reported two patterns of cognitive impairment in ECS individuals. Compared to HCs, ECS individuals showed significantly increased ALFF in the left caudate and bilateral thalamus and decreased ALFF in bilateral MPFC and bilateral PCC/PCu. Interestingly, we first found that ALFF of left PreCG and left PCC/PCu were different in the two identified impaired cognitive subtypes. In addition, ALFF of left PreCG and left PCC/PCu exhibited significant correlations with MCCB cognitive domains in ECS individuals. These findings suggest that the different spontaneous neural activities of PreCG and PCC/PCu at resting-state may be the potential neurobiological features of cognitive impairment subtypes in ECS.

Consistent with the grouping results of previous studies (Morar et al., 2011; Green et al., 2013), our LPA recognized two different patterns of cognitive impairment in ECS individuals. SI exhibited the worst performance in all MCCB cognitive scores, which manifested wide deficits in whole cognitive domains, ranging from 0.7 to 3.5 standard deviations below HCs (Figure 1 and Supplementary Table 2). MI showed an intermediate deficient speed of processing, attention/vigilance, verbal learning, visual learning, and social cognition, but maintained intact working memory and reasoning/problem solving, within ~1.2 standard deviations of HCs (Figure 1 and Supplementary Table 2).

In line with previous studies, a common phenomenon in both subtypes is that the speed of processing was the most severely impaired cognitive domain (η^2^ = 0.655, Table 2) except for the composite score. Speed of processing appears to be a core feature of cognition, it underlies other cognitive impairments such as executive functioning and working memory (Dickinson et al., 2007). Our study further supported that impaired speed of processing plays a key role in the cognitive impairment of ECS (Sheffield et al., 2018; Lim et al., 2020). Despite ECS individuals having a lower level of education, there was no difference in the educational years between the two impaired cognitive subtypes. Moreover, severely impaired individuals showed higher negative symptoms than moderately impaired individuals but with no differences in positive symptoms, which is consistent with the clinical observation that negative symptoms and cognitive impairment might share common pathophysiological substrates (Bowins, 2011; Lincoln et al., 2017).

Working memory is described as the ability to temporarily reserve and manipulate information for further cognitive processing (Baddeley, 1992). High-level cognitive processes require the support of working memory, such as reasoning, learning, and comprehension (Baddeley, 2003). The deficit in reasoning/problem-solving domain indicates executive dysfunction (Lis et al., 2005). Impaired executive function is found to predict poor functional outcomes, failure of interventions, and restricted recovery (Green et al., 2000). By using the LPA method, we identified a subtype with intact working memory and reasoning/problem solving from 155 ECS individuals, suggesting that executive function in the subtype with mild to moderate cognitive deficits is, indeed, comparable to the healthy population. In contrast to our study hypothesis, our results found only two cognitive subtypes. A possible reason is that due to our sample size limitation, it was not possible to cluster a subtype with a small proportion of ECS individuals. Another possible reason may be that our ECS individuals had a shorter duration and more severe psychiatric symptoms compared to other studies (Carruthers et al., 2019, 2021; Lim et al., 2020).

Before analyzing brain regions with ALFF differences in the two cognitive subtypes, we first examined regions with abnormal ALFF in ECS individuals. Resting-state functional MRI analyses showed four brain regions with abnormal neural activity in ECS, including two brain regions with increased ALFF (left caudate and bilateral thalamus) and two with decreased ALFF (bilateral MPFC and bilateral PCC/PCu).

Caudate is a part of the subcortical structure, which is responsible for several neurobiological processes such as planning the behavioral execution (Grahn et al., 2008). It has been found that there was hyperactivity in caudate during working memory tasks in individuals at clinical high risk for psychosis (Thermenos et al., 2016). Individuals with schizophrenia also exhibited higher ALFF in the left caudate than HCs (Zhang et al., 2021). Thalamus is also a subcortical region, which is involved in transmitting sensory information to the cerebral cortex and regulating emotion and cognitive attention control (Sherman, 2016; Wolff and Vann, 2019). Individuals with schizophrenia exhibited reduced thalamic gray matter volume (Alemán-Gómez et al., 2020), and abnormal activation during task-related functional MRI (Byne et al., 2009). As two parts of the cortico-striatal-thalamic-cortical (CSTC) sub-circuit in the salience network (SN), abnormal functional connectivity of caudate and thalamus has been reported in previous studies (Peters et al., 2016; Huang et al., 2020). Our findings showed that increased spontaneous neural activity appeared in the CSTC sub-circuit of the SN in the early course of schizophrenia, which may suggest a compensatory mechanism to maintain normal functioning performance (Gong et al., 2020). In addition, the long-term examination should be performed to investigate whether the changes in these two brain regions still exist.

Another important finding in our study is that ECS individuals showed decreased ALFF in bilateral MPFC and bilateral PCC/PCu compared with HCs. Both MPFC and PCC/PCu are core regions of the default mode network (DMN), a crucial brain network that associates with many neurophysiological functions (Raichle, 2015). The function of MPFC and PCC/PCu was involved in introspective processes that were attenuated when attention was turned to external events (Gusnard et al., 2001). There is increasing evidence that ECS individuals exhibited decreased ALFF in MPFC and PCC/PCu compared with HCs (Ren et al., 2013; Gong et al., 2020), which is in line with our results. Taken together, our findings further support that SN and DMN play a critical role in the pathogenesis of ECS.

Although we found ALFF abnormalities in several brain regions in ECS, not all the abnormalities could reflect the neuropathological changes in cognitive impairment. We found two regions with different ALFF values between SI and MI. Compared with MI, SI presented higher ALFF values in the left precentral gyrus (PreCG) and lower ALFF values in the left PCC/PCu. After controlling the PANSS total score and medication effects, these results remained significant. Moreover, ALFF of left PCC/PCu was positively correlated with MCCB cognitive scores in ECS, while ALFF of left PreCG showed negative correlations.

Several studies focused on PCC/PCu and cognitive function have been reported. A cortical morphometric study revealed that the structural volume of PCC/PCu was associated with cognitive impairment in first-episode schizophrenia (Wang et al., 2021). Furthermore, PCC/PCu showed significant activation during the episodic memory search task (Sestieri et al., 2011). In schizophrenia individuals, the activation in PCC/PCu was not significantly enhanced during the virtual maze task, while HCs exhibited significantly enhanced activation in the same region (Siemerkus et al., 2012). PreCG, also known as the primary motor cortex, is participated in motor information processing and emotional perception (Mesulam, 1998; Watanuki et al., 2016). Abnormal regional homogeneity and voxel-mirrored homotopic connectivity of PreCG have been found in first-episode schizophrenia (Liu et al., 2018a,b). Recent evidence confirmed that the excessive activity of PreCG could result in the impairment of emotional processing in schizophrenia (Watanuki et al., 2016). Additionally, another study indicated that PreCG might be involved in some cognitive processes, such as word recognition and phonological processing (Xu et al., 2019). Based on the available evidence, our results suggest that higher spontaneous neural activity in PCC/PCu is beneficial for the preservation of cognitive function in schizophrenia individuals, whereas higher activity in PreCG plays an opposite role. In conclusion, abnormal ALFF in caudate, thalamus, MPFC, and PCC/PCu reflected the pathophysiology of ECS, with abnormal ALFF in the PCC/PCu also indicating the pathophysiology of cognitive impairment in ECS. Furthermore, the abnormal spontaneous neural activity in the PCC/PCu in schizophrenia and between cognitive subtypes provides compelling evidence for the vital effect of DMN on schizophrenia and cognitive function.

Several limitations should be considered in our study. MRI data of subjects in our study were restricted by the fact that images were collected from different acquisition centers and scanners (Jovicich et al., 2006). We used the ComBat Harmonization method to minimize the site-specific confounds and enhanced statistical power (Fortin et al., 2017). However, the ComBAt Harmonization method cannot exclude the non-linear site and scanner effects, which is one of its drawbacks. For now, this method is still the best way to minimize linear site and scanner effects (Sun et al., 2022). Second, our sample size is relatively small, we propose to expand the sample size and make validation in the independent sample population in future. Third, our study is a cross-sectional study, and further follow-up study should be conducted to validate subtype stability and outcomes. Moreover, our study did not collect full-scale IQ from schizophrenia individuals and HCs, we will collect full-scale IQ in future studies, use the subtype classification based on MCCB to compare to the subtype classification based on full-scale IQ, and investigate the differences in full-scale IQ across cognitive subtypes.

In general, our results showed that cognitive impairment in ECS might be described as two subtypes: a severely impaired group with compromised cognition across all cognitive domains and a moderately impaired group with preserved cognition in working memory and reasoning/problem solving. Furthermore, our study identified four neurobiological features of ECS and two neurobiological features of cognitive subtypes in ECS. These brain regions associated with schizophrenia and cognitive function could be potential targets for the treatment of schizophrenia and its cognitive impairment. Meanwhile, differentiating individuals into subtypes based on cognitive function could help clinicians better understand the prognosis and recovery of social function, as well as carry out individualized interventions by combining the neuroimaging features derived from the subtypes. Our findings contribute to understanding the pathophysiology of cognitive impairment in ECS from the perspective of brain spontaneous neural activity.

## Data availability statement

The raw data supporting the conclusions of this article will be made available by the authors, without undue reservation.

## Ethics statement

The studies involving human participants were reviewed and approved by the Ethics Committee of the Second Xiangya Hospital of Central South University, the Ethics Committee of the Affiliated Nanjing Brain Hospital of Nanjing Medical University, the Ethics Committee of the First Affiliated Hospital of Zhengzhou University. Written informed consent to participate in this study was provided by the participants' legal guardian/next of kin.

## Author contributions

RW and JZ designed this study and wrote the protocol. RW, XS, XX, GH, YY, YL, XW, JX, YH, SG, JH, and YW performed the research. TS and WW analyzed the data and wrote the manuscript. RW, XS, and XX were involved in the revision and completion of the work. All authors contributed to the final manuscript.

## Funding

This work was supported by the National Natural Science Foundation of China (Grant Nos. 81622018 and 82072096).

## Conflict of interest

The authors declare that the research was conducted in the absence of any commercial or financial relationships that could be construed as a potential conflict of interest.

## Publisher's note

All claims expressed in this article are solely those of the authors and do not necessarily represent those of their affiliated organizations, or those of the publisher, the editors and the reviewers. Any product that may be evaluated in this article, or claim that may be made by its manufacturer, is not guaranteed or endorsed by the publisher.
