# Supplementary material for: Identifying and revealing different brain neural activities of cognitive subtypes in early course schizophrenia
Source: Front Mol Neurosci. 2022 Oct 3;15:983995. doi: 10.3389/fnmol.2022.983995 (PMC9577612; doi:10.3389/fnmol.2022.983995)
Supplement: Supplementary file 1 [file Data_Sheet_1.docx]

Supplementary Material

**1 Supplementary Methods**

**1.1 Neuroimaging data acquisition**

All the magnetic resonance images were collected from the three centers mentioned in the main text, including structural image data using high-resolution T1-weighted 3D imaging and resting-state functional image data using gradient echo-planar imaging (EPI). We used a standard 8-channel head coil capping on a participant’s head to receive magnetic resonance signals. Before the MRI scanning, earplugs and foam earmuffs were used to reduce noise and restrict head motion during scanning. Participants were required to remain still and awake with their eyes closed. The MRI scanning parameters of each center are as follows.

Center 1*.* The magnetic resonance images from Center 1 were acquired on a Philips 3T scanner (Philips Healthcare, Best, The Netherlands). T1-weighted images were acquired with these parameters: repetition time (TR) = 8.25 ms, echo time (TE) = 3.78 ms, inversion time (TI) = 1100 ms, flip angle (FA) = 7°, matrix size = 256 × 256, field of view (FOV) = 256 mm × 256 mm, slice thickness = 1 mm, no gap and 188 sagittal slices. The parameters of EPI sequence were as follows: TR = 2000 ms, TE= 30 ms, FA = 90°, matrix size = 64 × 64, FOV = 220 mm × 220 mm slice thickness = 4.0 mm, 33 transverse slices and 240 volumes.

Center 2*.* MRI images of Center 2 were performed on a Siemens 3T system (Siemens, Erlangen, Germany). T1-weighted images were collected with these following parameters: TR = 2530 ms, TE = 3.45 ms, TI = 1100 ms, FA= 7°, matrix size = 256 × 256, FOV = 256 mm × 256 mm, slice thickness = 1 mm, no gap and 192 sagittal slices. Functional images were acquired with the parameters as follows: TR = 2000 ms, TE = 30 ms, FA = 90°, matrix size =64×64, FOV = 220 mm × 220 mm, slice thickness = 4.0 mm, 33 transverse slices and 240 volumes.

Center 3. All MRI scans were conducted using a General Electric 3T scanner (General Electric, Milwaukee, Wisconsin, USA). T1-weighted images were acquired using a brain volume sequence with following parameters: slice thickness = 1mm, 188 sagittal slices, TR = 8.17 ms, TE = 3.18 ms, TI = 450 ms, FA = 12°, matrix size = 256 × 256 and FOV = 256 mm × 256 mm. Functional images were acquired with the following parameters: TR = 2000 ms, TE = 30 ms, FA = 90°, matrix size =64×64, FOV = 220 mm × 220 mm, slice thickness = 4.0 mm, 32 transverse slices and 180 volumes.

**2 Supplementary Results**

**2.1 ALFF differences between cognitive subtypes (controlled PANSS total score and medication effects)**

We controlled age, sex, education, mean frame-wise displacement Jenkinson, PANSS total score and medication effects (chlorpromazine dose equivalence) for this extra comparison. The results were consistent with and without PANSS total score and medication effects as covariates (Supplementary Table 3).

**3 Supplementary Figures and Tables**

**3.1 Supplementary Figures**


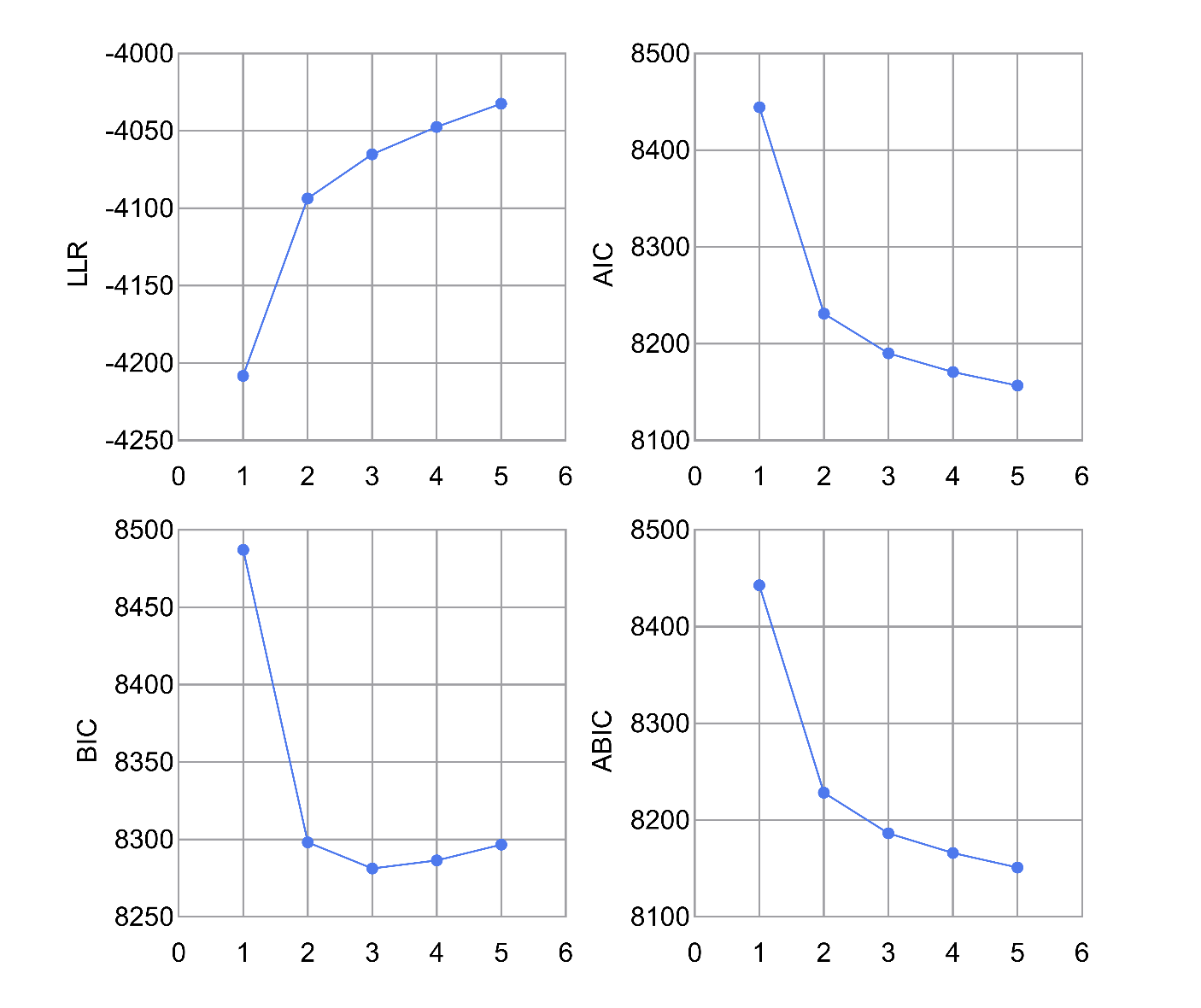
**Supplementary Figure 1.** Scree plots for latent profile analysis. Y-axis represents information criteria value for each plot (e.g., AIC value). The X-axis represents the number of classes. *Abbreviations:* LLR, log-likelihood ratio; AIC, Akaike’s information criteria; BIC, Bayesian information criteria; ABIC, sample-size adjusted Bayesian information criteria.

**3.2 Supplementary Tables**

| **Table S1.** Demographic and clinical data of ECS cognitive subtypes (SI and MI) and HCs of each center. | | | | | | | | | | | | |  |
| --- | --- | --- | --- | --- | --- | --- | --- | --- | --- | --- | --- | --- | --- |
|  | Center 1 | | |  | Center 2 | | |  | Center 3 | | |  | |
|  | SI  (*n* = 9) | MI  (*n* = 25) | HC  (*n* = 18) |  | SI  (*n* = 7) | MI  (*n* = 31) | HC  (*n* = 36) |  | SI  (*n* = 47) | MI  (*n* = 36) | HC  (*n* = 43) |  | |
| Age, years | 22.89 (5.33) | 24.80 (8.09) | 24.00 (6.81) |  | 28.57 (12.08) | 29.52 (9.16) | 28.58 (6.39) |  | 23.32 (6.27) | 22.22 (6.31) | 22.49 (1.53) |  | |
| Sex, female/male | 6/3 | 17/8 | 11/7 |  | 5/2 | 17/14 | 20/16 |  | 15/32 | 25/11 | 23/20 |  | |
| Education, years | 10.00 (1.50) | 11.24 (2.93) | 12.27 (2.46) |  | 13.43 (3.51) | 12.90 (3.18) | 16.06 (2.15) |  | 11.15 (2.41) | 10.00 (2.10) | 10.79 (2.07) |  | |
| Duration, months | 19.11 (12.54) | 10.08 (9.88) | - |  | 21.72 (13.87) | 16.26 (12.10) | - |  | 3.95 (3.92) | 4.78 (4.13) | - |  | |
| CPZ-DDD, mg | 293.33 (81.85) | 259.20 (54.00) |  |  | 305.71 (153.50) | 337.74 (195.00) |  |  | 240.00 (0.00) | 240.00 (0.0) |  |  | |
| PANSS |  |  |  |  |  |  |  |  |  |  |  |  | |
| Positive symptoms | 18.44 (5.96) | 16.84 (5.65) | - |  | 25.00 (7.26) | 26.45 (4.80) | - |  | 24.09 (4.55) | 21.50 (4.89) | - |  | |
| Negative symptoms | 29.44 (6.67) | 22.32 (5.35) | - |  | 28.57 (8.50) | 21.23 (7.37) | - |  | 26.38 (5.62) | 23.08 (4.80) | - |  | |
| General psychopathology | 41.78 (8.60) | 42.72 (4.70) | - |  | 47.43 (8.18) | 44.61 (7.98) | - |  | 47.40 (5.99) | 44.11 (5.10) | - |  | |
| Total score | 89.67 (16.06) | 81.88 (9.43) | - |  | 101.00 (16.50) | 92.26 (14.85) | - |  | 97.87 (11.84) | 89.23 (10.72) | - |  | |
| MCCB |  |  |  |  |  |  |  |  |  |  |  |  | |
| Speed of processing | 23.48 (6.50) | 38.93 (5.37) | 50.17 (7.74) |  | 22.81 (8.08) | 40.02 (7.29) | 45.45 (7.03) |  | 19.74 (7.85) | 35.25 (7.51) | 46.05 (7.22) |  | |
| Attention/vigilance | 33.00 (12.67) | 46.96 (12.34) | 51.72 (9.70) |  | 29.57 (11.79) | 38.16 (9.81) | 44.19 (8.66) |  | 23.47 (10.09) | 37.14 (8.44) | 50.86 (8.32) |  | |
| Working memory | 27.00 (8.23) | 41.84 (8.37) | 44.78 (9.15) |  | 28.57 (13.33) | 43.31 (10.10) | 32.42 (11.55) |  | 33.68 (9.25) | 43.31 (10.10) | 47.21 (9.90) |  | |
| Verbal learning | 23.00 (9.21) | 38.28 (8.00) | 45.83 (11.66) |  | 16.14 (7.40) | 42.45 (9.33) | 43.33 (10.24) |  | 30.15 (6.7) | 43.50 (7.85) | 44.35 (7.11) |  | |
| Visual learning | 26.78 (14.52) | 41.20 (12.77) | 53.22 (8.72) |  | 22.57 (8.18) | 42.26 (8.82) | 49.00 (12.53) |  | 29.55 (13.76) | 48.28 (8.38) | 46.72 (10.12) |  | |
| Reasoning/problem solving | 28.44 (10.99) | 40.24 (15.27) | 49.33 (9.19) |  | 28.00 (10.46) | 41.42 (11.13) | 42.97 (11.03) |  | 29.09 (6.34) | 38.25 (9.00) | 36.70 (8.44) |  | |
| Social cognition | 29.89 (9.48) | 41.44 (10.89) | 52.28 (6.67) |  | 41.57 (10.81) | 40.87 (10.48) | 50.86 (8.47) |  | 29.02 (8.51) | 37.42 (11.03) | 40.33 (8.96) |  | |
| Composite score | 26.51 (4.52) | 40.75 (5.80) | 49.74 (4.87) |  | 26.10 (7.49) | 40.34 (4.47) | 44.35 (6.27) |  | 26.02 (4.62) | 39.29 (4.56) | 44.92 (4.59) |  | |
| Values are presented as mean (SD). SI and MI represent the two cognitive subtypes in ECS. *Abbreviations:* ECS, early course schizophrenia; SI, severely impaired subtype; MI, moderately impaired subtype; HC, healthy control; CPZ-DDD, chlorpromazine-defined daily dose; MCCB, MATRICS Consensus Cognitive Battery; PANSS, Positive and Negative Syndrome Scale; SD, standard deviation. | | | | | | | | | | | | |  |

| **Table S2.** MCCB scores of ECS cognitive subtypes (SI and MI) standardized against HC values. | | | |
| --- | --- | --- | --- |
| MCCB | SI (*n* = 63) | MI (*n* = 92) | HC (*n* = 97) |
| Speed of processing | -3.52 (1.05) | -1.18 (0.97) | 0.00 (1.00) |
| Attention/vigilance | -2.49 (1.20) | -0.91 (1.17) | 0.00 (1.00) |
| Working memory | -0.73 (0.79) | -0.02 (0.76) | 0.00 (1.00) |
| Verbal learning | -1.81 (0.90) | -0.27 (0.93) | 0.00 (1.00) |
| Visual learning | -1.85 (1.22) | -0.40 (0.93) | 0.00 (1.00) |
| Reasoning/problem solving | -1.18 (0.70) | -0.14 (1.09) | 0.00 (1.00) |
| Social cognition | -1.60 (0.96) | -0.68 (1.09) | 0.00 (1.00) |
| Composite score | -3.46 (0.87) | -0.99 (0.87) | 0.00 (1.00) |
| Values are presented as mean (SD). *Abbreviations:* ECS, early course schizophrenia; SI, severely impaired subtype; MI, moderately impaired subtype; HC, healthy control; MCCB, MATRICS Consensus Cognitive Battery; SD, standard deviation. | | | |

| **Table S3.** Brain regions with ALFF differences in ECS cognitive subtypes (controlled PANSS total score and medication effects). | | | | | | |
| --- | --- | --- | --- | --- | --- | --- |
|  | Brain regions | Cluster size | Peak coordinate (mm)^a^ | | | Peak *t*-value |
|  |  |  | x | y | z |  |
| SI > MI | PreCG/IFGoper L | 61 | -54 | 3 | 18 | 5.2209 |
| SI < MI | PCC/PCu L | 57 | -9 | -54 | 24 | -4.3479 |
| SI and MI represent the two cognitive subtypes in ECS.  ^a^ Peak coordinate refers to the peak voxel location of the significant cluster in the Montreal Neurological Institute space.  *Abbreviations:* ALFF, amplitude of low-frequency fluctuations; ECS, early course schizophrenia; SI, severely impaired subtype; MI, moderately impaired subtype; PANSS, Positive and Negative Syndrome Scale; PreCG, precentral gyrus; IFGoper, inferior frontal gyrus-opercular part; PCC/PCu, posterior cingulate cortex/precuneus; L, left. | | | | | | |
